# Rigorous Plasma Microbiome Analysis Method Enables Disease Association Discovery in Clinic

**DOI:** 10.3389/fmicb.2020.613268

**Published:** 2021-01-08

**Authors:** Zhenwu Luo, Alexander V. Alekseyenko, Elizabeth Ogunrinde, Min Li, Quan-Zhen Li, Lei Huang, Betty P. Tsao, Diane L. Kamen, Jim C. Oates, Zihai Li, Gary S. Gilkeson, Wei Jiang

**Affiliations:** ^1^Department of Microbiology and Immunology, Medical University of South Carolina, Charleston, SC, United States; ^2^Program for Human Microbiome Research, Biomedical Informatics Center, Department of Public Health Sciences, Medical University of South Carolina, Charleston, SC, United States; ^3^Department of Oral Health Sciences and Department of Healthcare Leadership and Management, Medical University of South Carolina, Charleston, SC, United States; ^4^Department of Immunology and Internal Medicine, University of Texas Southwestern Medical Center, Dallas, TX, United States; ^5^Treatment and Research Center for Infectious Diseases, The Fifth Medical Center of Chinese PLA General Hospital, Beijing, China; ^6^Division of Rheumatology and Immunology, Department of Medicine, Medical University of South Carolina, Charleston, SC, United States; ^7^Ralph H. Johnson VA Medical Center, Medical Service, Charleston, SC, United States; ^8^Pelotonia Institute for Immuno-Oncology, The Ohio State University Comprehensive Cancer Center-James, Columbus, OH, United States; ^9^Division of Infectious Diseases, Department of Medicine, Medical University of South Carolina, Charleston, SC, United States

**Keywords:** plasma microbiome, quality-filtering strategies, contaminations and artifacts, pathogenic bacteria, microbiome-host interaction

## Abstract

Blood microbiome is important to investigate microbial-host interactions and the effects on systemic immune perturbations. However, this effort has met with major challenges due to low microbial biomass and background artifacts. In the current study, microbial 16S DNA sequencing was applied to analyze plasma microbiome. We have developed a quality-filtering strategy to evaluate and exclude low levels of microbial sequences, potential contaminations, and artifacts from plasma microbial 16S DNA sequencing analyses. Furthermore, we have applied our technique in three cohorts, including tobacco-smokers, HIV-infected individuals, and individuals with systemic lupus erythematosus (SLE), as well as corresponding controls. More than 97% of total sequence data was removed using stringent quality-filtering strategy analyses; those removed amplicon sequence variants (ASVs) were low levels of microbial sequences, contaminations, and artifacts. The specifically enriched pathobiont bacterial ASVs have been identified in plasmas from tobacco-smokers, HIV-infected individuals, and individuals with SLE but not from control subjects. The associations between these ASVs and disease pathogenesis were demonstrated. The pathologic activities of some identified bacteria were further verified *in vitro*. We present a quality-filtering strategy to identify pathogenesis-associated plasma microbiome. Our approach provides a method for studying the diagnosis of subclinical microbial infection as well as for understanding the roles of microbiome-host interaction in disease pathogenesis.

## Introduction

Blood and tissues were originally presumed to be sterile, and microbes were thought to occur only in cases of sepsis and live bacterial infections. However, recent research evidence has shown live bacteria in the blood and tissues which may play a role in the disease pathogenesis. Poore’s group found cancer-specific microbial sequences in the tissues and the blood from different types of cancer in humans (Poore et al., 2020). In addition, another study led by Nejman’s group found that distinct microbiome composition in seven cancer types and that the bacteria localized within both tumors and immune cells (Nejman et al., 2020). Microbe or microbial components were found in the blood from individuals with chronic inflammatory diseases (Potgieter et al., 2015; Lelouvier et al., 2016; Luo et al., 2019). Using bacterial 16S rRNA sequencing, Massier’s group showed bacteria in the blood and adipose tissue samples, which were associated with increased tissue inflammation in obesity and type 2 diabetes (Massier et al., 2020). Moreover, the translocated microbial products were shown to induce immune perturbations and may contribute to some disease immunopathogenesis such as autoimmune diseases and central nervous system diseases (Niebauer et al., 1999; Brenchley et al., 2006; Klatt et al., 2010; Yoshimoto et al., 2013; Costa et al., 2016; Vieira et al., 2018). Our recent study showed that translocation of *Staphylococcus* promotes germinal center B cell activation and autoantibody production in mice and HIV+ individuals (Luo et al., 2019). All of these studies indicated that plasma or tissue microbiome might contribute to immune perturbations and disease pathogenesis.

However, investigation of plasma or tissue microbiome is highly challenging because of extremely low levels of bacterial biomass in the blood or tissues under a physiological condition, which leads to a high risk of contamination during procedures from plasma microbial cell-free DNA (cfDNA) isolation, amplification, to sequencing. Using whole-genome and whole-transcriptome sequencing, up to 92.3% of plasma microbiome sequence data was discarded after stringent decontamination to ensure valid results (Poore et al., 2020). Therefore, removing the background and artifacts from sequencing data is critical to obtain accurate results from the plasma microbiome analysis.

In this study, we applied a quality-filtering strategy to plasma microbiome to efficiently exclude amplicon sequence variants (ASVs) of contaminations and artifacts. After strict controls of contamination during plasma microbial DNA isolation, we found more than 97% of plasma microbial 16S DNA sequencing data resulted from low abundance and low prevalence of microbial sequences, contaminations, and artifacts, which was removed using stringent quality-filtering strategy analyses. Furthermore, we analyzed plasma microbiome in three cohorts, including tobacco-smoking individuals, individuals with HIV infection, or systemic lupus erythematosus (SLE), as well as corresponding control individuals.

## Materials and Methods

### Subjects

Volunteers with SLE, HIV-infected individuals, and healthy individuals were recruited from the Medical University of South Carolina (MUSC) Lupus Clinic, Clinic of Infectious Diseases, and MUSC campus. The clinical characteristics are shown in Supplementary Table S1. All individuals with SLE met at least four American College of Rheumatology criteria for the classification of lupus as determined by the rheumatologist (Hochberg, 1997). The SLE clinical characteristics, disease manifestations, and medications being used are shown in Supplementary Table S2. HIV-infected individuals have been treated with antiretroviral therapy (ART) and have plasma HIV RNA below the limit of detection for at least 24 weeks (a single blip to ≤500 copies/ml was allowed). Tobacco-smoking and non-smoking control individuals were recruited from the Fifth Medical Center of Chinese PLA General Hospital in China. All tobacco-smokers were smoking for at least 5 years. This study was approved by each of the participating institutional review boards. All participants provided written informed consent. The tobacco-smoking cohort included 20 tobacco smokers and 21 non-smokers; the HIV cohort included 40 aviremic ART-treated HIV-infected individuals and 51 healthy controls; the SLE cohort included 19 women with SLE and 30 healthy control women. Venous blood was drawn into EDTA tubes and unstimulated saliva was collected after rinsing the mouth following a standard protocol (Topkas et al., 2012).

### Plasma Microbial 16S rDNA Isolation

Fresh blood samples or endotoxin-free water (negative control, Catalog number: W50-640, LONZA, Walkersville, MD, United States) in EDTA-containing tubes (BD, San Jose, CA, United States) were centrifuged at 800 *g* for 15min, which was followed by transferring the samples to new centrifuge tubes (Catalog number: 352098, BD). Plasma and water controls were placed in aliquots and stored at −80°C. DNA low-binding centrifuge tubes (Catalog number: 022431021, Eppendorf, Hamburg, Germany) were used to store the plasma samples. We avoided repeated freezing and thawing before microbial 16S rDNA isolation. Circulating bacterial DNA was extracted from 400 μl of plasma or the water control using the QIAamp UCP Pathogen Mini Kit (Catalog number: 50214, Qiagen, United States) according to the manufacturer’s instructions.

### 16S rDNA Sequencing

Bacteria 16S rDNA was isolated from plasma and amplified using primers 515/806 with the barcode on the forward primer using a 35-cycle PCR. The HotStarTaq Plus Master Mix Kit (Catalog number: 203645, Qiagen) was used, and the 16S V4 variable region was amplified. To prevent batch-to-batch variation, all samples in each cohort were run concurrently. The PCR was performed under the following conditions: 94°C for 3 min, followed by 35 cycles at 94°C for 30 s, 53°C for 40 s and 72°C for 1 min, and a final elongation step at 72°C for 5 min. The PCR products were checked on a 2% agarose gel to assess the amplification. Multiple samples were pooled together in equal concentrations and purified using Agencourt AMPure XP beads (Catalog number: A63880, Beckman Coulter, Brea, CA, United States). The pooled and purified PCR products were used to prepare the DNA library according to the Illumina TruSeq DNA library preparation protocol. Sequencing was performed at MR DNA (www.mrdnalab.com, Shallowater, TX, United States) on a MiSeq platform following the manufacturer’s guidelines.

### Microbial 16S rDNA Data Processing

QIIME2^1^ was applied to demultiplex the data generated by Illumina MiSeq sequencing into paired forward and reverse FASTQ. For each sample, barcodes and primers were depleted at paired sequences. Demultiplexed sequences were processed using the DADA2 (version 1.8; Callahan et al., 2016a) analysis pipeline in the R (https://www.r-project.org/, version 3.5.0) environment. Briefly, paired reads likely contain low quality throughout and pathological errors were truncated, and more than two expected errors per read were removed by paired reads (Edgar and Flyvbjerg, 2015). Unsupervised learning in DADA2 was performed to distinguish sequencing errors from real biological variation, while replacement and insertion of errors from the data were removed at the inference step. After merging the paired sequences, the chimera sequences were removed. ASVs were defined as clustering at 1% divergence (99% similarity), and the naive Bayesian classifier method was applied to assign taxonomy using the RDP v16 training set (Callahan et al., 2016b).

### Plasma 16S Microbiome Analysis

ASV tables and different levels of taxonomic tables were imported into a phyloseq R package (version 3.7; McMurdie and Holmes, 2013) for statistical analysis. A 0.005% minimum abundance threshold was applied to exclude ASVs with a low abundance. Next, a prevalence filter was applied to ASVs, and the ASVs were retained only if the prevalence of specific ASV was more than 1/3 in any study group samples. A user-defined control filtering factor was applied to remove the potential contaminants and artifacts from the plasma microbiome. If the abundance of taxa from blank controls multiplied by the filtering factor was more than the abundance of taxa from plasma samples, the taxa were removed from samples. *α*-diversity of richness and evenness was calculated by the Simpson index of diversity in each sample. The Unifrac coefficient was calculated to evaluate *β*-diversity and compositional dissimilarity among the microbial community. The Multivariate Welch *t*-test was used to test the statistical significance of variances in microbiome composition between groups (Alekseyenko, 2016). Differential abundance testing between groups was compared by nonparametric Mann-Whitney’s *U* tests at the OTU level. Values of *p* were adjusted for multiple comparisons by the Benjamin-Hochberg false discovery rate (FDR). The comparison analysis was performed using R.

### Autoantigen Microarray

Plasma levels of IgG autoantibodies were analyzed using 125-plex autoantigen arrays at the Genomics and Microarray Core, University of Texas Southwestern Medical Center. Plasma samples were treated with DNase I, diluted 1:50, and incubated with the autoantigen arrays. Cy3-labeled anti-human IgG was used to detect IgG autoantibodies. Mean fluorescence intensities (MFI) represent the signal intensity of each autoantibody.

### Pro-inflammatory Cytokine Induction Stimulated by Disease-Enriched Bacteria *in vitro*

Peripheral blood mononuclear cells (PBMCs) from healthy individuals were isolated over a Ficoll-Hypaque cushion (GE, Pittsburgh, PA). After the bacteria were heat-inactivated at 60°C for 30 min, *Massilia timonae*, *Haemophilus parainfluenzae*, and *Anaerococcus prevotii* were added to cell culture at a final concentration of 5 × 10^6^/ml; LPS from *Escherichia coli* 055:B5 was used as a positive control with a final concentration of 2 μg/ml. PBMCs were cultured with brefeldin A (5 μg/ml, BD) and incubated at 37°C for 6 h. Cells were then collected and washed with PBS, followed by a 20-min incubation with 50 μl aqua blue (Life Technologies, Carlsbad, CA, United States) at 4°C to exclude dead cells. Next, 50 μl of an antibody cocktail containing anti-human CD3 (OKT3), anti-human CD14 (M5E2), and anti-human CD16 (3G8) were used for surface staining. After washing and permeabilization, cells were intracellularly stained with anti-human TNF-α (MAb11), anti-human IL-1β (AS10), and anti-human IL-6 (MQ2-6A3). Fluorescence-labeled antibodies were purchased from BD or Biolegend (San Diego, CA). After washing, cells were collected and analyzed using a BD FACSVerse flow cytometer (BD). Data were analyzed using the FlowJo software (version 10.0.8).

## Results

### 16S rDNA Plasma Microbiome Analysis

Unlike saliva or feces, plasma has extremely low levels of bacterial DNA under a physiological condition but not a live infection. The external introduction of microbes or microbial DNAs through reagents and experimental procedures may cause artifacts in the plasma microbiome analyses. To study the source of potential artifacts, we investigated possible contaminations through the procedures of drawing blood, cfDNA isolation, and sequencing. Several controls were established to evaluate contaminations (Supplementary Figure S1A), as follows: (1) control i was the water control sequenced directly without cfDNA isolation; (2) control ii was the water control sequenced in isolated cfDNA; (3) control iii was the water control obtained through mimicking the whole procedure of drawing blood and plasma collection, cfDNA isolation, and sequencing. For this control, the skin of the antecubital fossa was wiped with 75% ethanol, and the sterile blood collection needle was used to puncture the skin but not into blood vessel. Next, the needle was withdrawn from the skin and, instead of drawing blood, the water in the eppendrof tubes will be drawn into blood collection tubes; and (4) control iv was the water control used to assess possible contamination from the skin microbiota, for which the skin in the cubital fossa was wiped with 75% ethanol over areas of 3–4 cm^2^ and allowed to dry. We added water to the “clean” skin, collected the water, and isolated and sequenced microbial cfDNA.

We first isolated microbial cfDNA from plasma of 25 healthy individuals. After 16 s rDNA sequencing, we performed the following steps: filtering and trimming of low-quality sequences, dereplication, merging of paired reads, and removal of chimeras. To determine whether the overall microbiome composition differed according to 16S isolation and sequencing, as well as within sample diversity (*β*-diversity), we conducted principal coordinate analysis (PCoA) based on unweighted UniFrac phylogenetic distances. There was no difference in the *β*-diversity of water control i, ii, and iii. However, the 16S rDNA isolated from control iv, obtained from the skin surface, showed differences in diversity when compared with the water controls i, ii, and iii (*p* < 0.001, Multivariate Welch *t*-test; Supplementary Figure S1B). Notably, the β-diversity of plasma microbial 16S rDNA was significantly different from those of the water controls (*p* < 0.001; Supplementary Figure S1B). This finding indicates that most artifacts occurred from the PCR and sequencing process. Although microbial 16S sequences in the water controls had backgrounds or contaminants, they were different from the 16S sequences of the plasma microbiome.

### Removing Potential Artifacts and Contaminants From Plasma Microbiome

**Figure 1:**
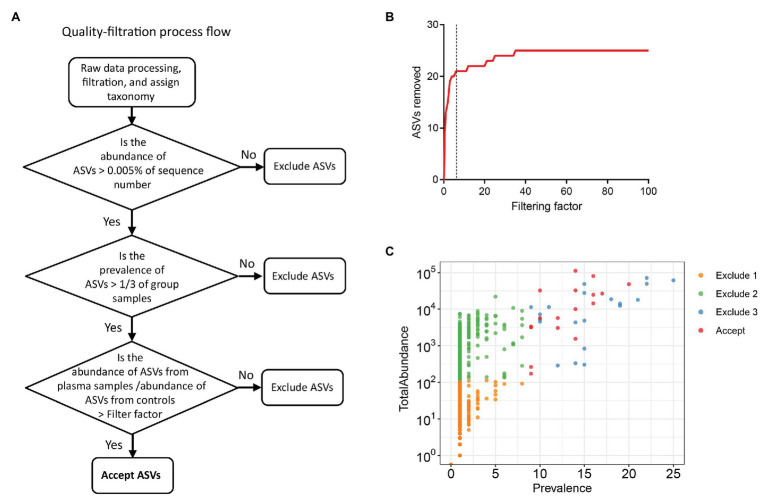
Strategies for removing background and potential artifacts from plasma microbiome. **(A)** A workflow diagram of user-defined quality-filtering strategy to exclude the background and artifacts. **(B)** The number of removed amplicon sequence variants (ASVs) with different filtering factors; we chose the filtering factor of 6 which showed the lowest number of the plateau effect. **(C)** The abundance and prevalence of ASVs in each step of filtration. The “exclude 1” step is removing low abundance of ASVs across samples, in which a 0.005% minimum abundance threshold was applied. The “exclude 2” step is removing low prevalence of ASVs in each sample; we retained ASVs only if its prevalence was more than 1/3 across study groups. The “exclude 3” step is removing potential contaminants and artifacts using filtering factors.

To exclude possible contaminant sequences and artifacts in the analysis, we performed a user-defined quality-filtering strategy (Figure 1A). We chose control iii as the blank control because it reflected the entire specimen handling process. A challenge for plasma microbiome analysis is the presence of heavy tail distribution and low prevalence of ASVs across samples. Therefore, before the removal of artifacts observed in plasma microbiome, a 0.005% minimum abundance threshold was applied to exclude ASVs with low abundance (Bokulich et al., 2013). In this case, ASVs with an abundance of less than 5 in each sample were removed; thus, 547 ASVs were excluded and 568 ASVs were retained after filtration. The prevalence of ASVs in plasma microbiome of each donor may vary dramatically due to factors such as donor differences in the local microbiota communities and translocation from mucosal sites (e.g., oral cavity, gut, or vagina). Thus, to study the function of plasma microbiome in disease etiology or pathogenesis, it is essential to remove the interference from ASVs, which may exhibit a high abundance in some samples but a low prevalence across samples. To study the associations between enriched blood microbiome and disease pathogenesis, we excluded the ASVs shown in less than 1/3 individuals in either control or studied disease group (prevalence less than 1/3). After removing the low prevalence of ASVs, 527 ASVs were excluded and 41 remained.

Excluding all ASVs that appear in the controls may result in the loss of meaningful data, a filtering factor was applied to help ascertain whether the ASVs found in the control were due to cross-contamination from a real sample or from contaminations or artifacts by reagents during microbial DNA isolation and sequencing. If the taxa abundance in the water controls multiplied by the filtering factor was more than the taxa abundance in the plasma samples, the taxa present in the water control were considered contaminations from reagents or overamplification; thus, they were removed from the sample analyses. Otherwise, the taxa in the water control were considered cross-contamination from the plasma sample, and the taxa were retained. Figure 1B shows the number of removed ASVs when different filtering factors were applied. To exclude potential contaminants without loss of authentic information, we chose the filtering factor of six which showed the lowest number of the plateau effect (Figure 1B). A total of 16 ASVs were considered artifacts and removed from the samples, and 25 ASVs were accepted after filtration. The filtered-out ASVs from plasma microbiome varied from low to high abundance across the samples (Figure 1C).

### Comparisons Between Plasma and Saliva Microbiome in Tobacco Smokers

**Figure 2:**
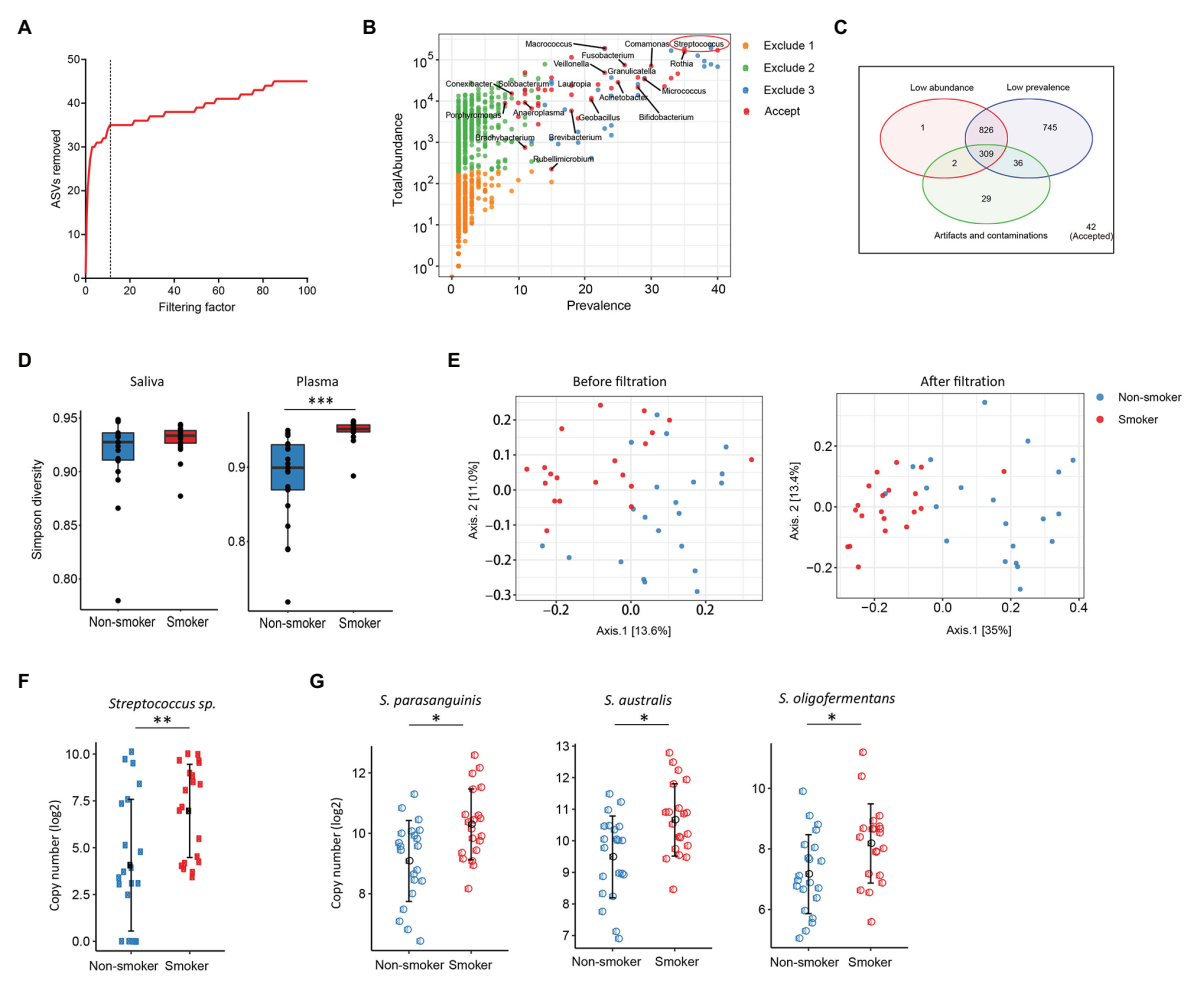
Plasma and oral microbiome analyses in tobacco smokers compared with non-smokers. **(A)** The number of removed ASVs from plasma microbiome with different filtering factors; we chose the filtering factor of 11 which showed the lowest number of the plateau effect. **(B)** The abundance and prevalence of ASVs in each step of plasma microbiome filtration. The significantly different taxa in smokers compared with those in non-smokers are labeled in black (enriched in smokers). **(C)** Venn diagram showing the number and overlap of low abundance, low prevalence, and contaminations/artifacts in plasma microbiome. **(D)** The Gini Simpson diversity index (*α*-diversity) was used to compare the diversity of oral and plasma microbiome between smokers and non-smokers after filtration. **(E)** Principal coordinate analysis (PCoA) was conducted based on the unweighted UniFrac distance to determine the beta diversity of plasma microbiome before and after filtration. **(F)** *Streptococcus* was enriched in plasma microbiome of smokers compared to non-smokers. **(G)** Representative *Streptococcus* species in the oral microbiome of smokers and non-smokers. Mann-Whitney *U* (unpaired) and Spearman’s rank tests. ^*^p < 0.05, ^**^p < 0.01, ^***^p < 0.001.

Various disease states or altered microbiota composition may lead to a compromised mucosal epithelial barrier and translocation of microbial products into circulation. The use of tobacco is associated with the changes of oral microbiota and leads to a higher rate of periodontal diseases (Thomson et al., 2008). Translocation of oral bacteria into the blood has been implicated in the development of myocarditis, endocarditis, and cerebral infarction (Koren et al., 2011; Seringec et al., 2015). To investigate whether plasma microbiome is changed along with the mucosal microbiota, we have analyzed and compared the plasma and saliva microbiome from tobacco smokers and non-smoker controls. Blood and saliva samples were collected simultaneously for microbial 16S sequencing in 21 non-smokers and 20 tobacco smokers. In the saliva microbiome, we obtained 695 ASVs from 16S sequencing; after removing the ASVs with low abundance, 119 ASVs were retained from a total of 41 participants. In the plasma samples, as described above, after removing the ASVs with extremely low abundance and low prevalence, we removed potential contaminants using a filtering factor of 11 which was the lowest number of the plateau effect (Figure 2A); 42 ASVs were retained from 1,989 ASVs (2.1%) in 41 participants (Figures 2B,C).

There was no difference in the species diversity within each sample in the saliva from smokers and non-smokers based on the Gini Simpson (α-diversity) index (Figure 2D). However, the alpha-diversity of plasma microbiome in smokers was significantly higher compared with that of non-smokers (Figure 2D), suggesting that smoking may alter the oral microenvironment directly or indirectly through host immune responses to microbiota. In addition, unweighted UniFrac phylogenetic distances showed that tobacco smoking significantly altered the overall circulating microbiome composition (Figure 2E). In plasma samples, before removing the contaminants and ASVs with low prevalence, Axis 1 and Axis 2 of the PCoA interpreted 13.6 and 11% of the difference within samples, respectively; after filtering, Axis 1 and Axis 2 of the PCoA interpreted 35 and 13.4% of the difference within samples, respectively (Figure 2E).

To analyze the components of ASVs in the smoking and non-smoking groups, Benjamini and Hochberg FDR corrections were employed to adjust for multiple comparisons after performing nonparametric Mann-Whitney’s *U* tests. In saliva samples, 25 ASVs were significantly different in smokers compared to non-smokers before adjusting for multiple comparisons, including 18 ASVs increased and 7 ASVs decreased in smokers compared to non-smokers (Supplementary Table S3). In plasma samples, 20 ASVs were different in smokers compared to healthy controls after adjusting with the FDR, and all taxa identified were enriched in the smokers (Supplementary Table S4). Among the taxa that were increased in the saliva of tobacco smokers, eight belonged to the *Streptococcus* family (Supplementary Table S3), which was also significantly enriched in the plasma microbiome of tobacco smokers (Figures 2F,G). *Streptococcus* was shown enrichment in both plasma and saliva from smokers and non-smokers, implying that some oral bacteria or bacterial products may translocate to the circulation. The shared microbiome in plasma and saliva could be a result of increased absolute numbers of specific microbiome or a preferential migration ability of certain taxa from altered oral or periodontal environment to the circulation in smokers.

### Plasma Microbiome in HIV Disease

**Figure 3:**
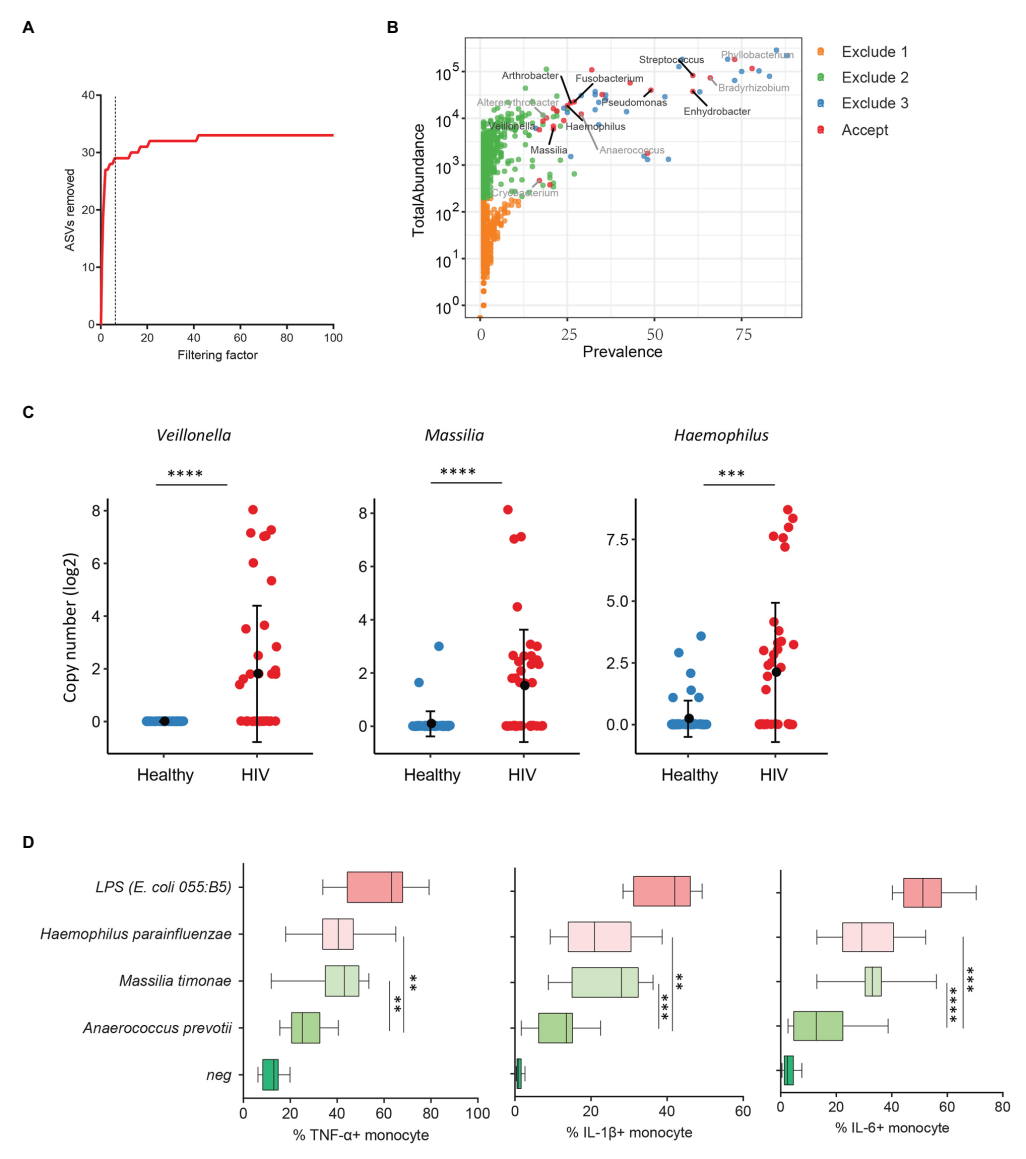
Plasma microbiome in aviremic antiretroviral therapy (ART)-treated HIV+ individuals and healthy controls. **(A)** The number of removed ASVs with different filtering factors; we chose the filtering factor of 6 which showed the lowest number of the plateau effect. **(B)** The abundance and prevalence of ASVs in each step of plasma microbiome filtration are shown, and the significantly different taxa in HIV+ individuals compared with healthy controls are labeled in black (enriched in HIV+ individuals) or gray (decreased in HIV+ individuals). **(C)** The detected copies of the top three most increased taxa were shown in HIV+ individuals compared with healthy controls. **(D)** The percentages of TNF-α, IL-1β, and IL-6-producing monocytes in total monocytes were shown after peripheral blood mononuclear cells (PBMCs) were stimulated with heat-inactivated bacteria (5 × 10^6^ units/ml) or 2 μg/ml of LPS (*n* = 15, healthy individuals). Cytokines were measured in monocytes using flow cytometry after 6 h of stimulation. Box and whisker plots (Min. to Max.) show the responses of monocytes to each bacterium or LPS. Nonparametric Mann-Whitney’s *U* tests and one-way ANOVA test. ^*^p < 0.05, ^**^p < 0.01, ^***^p < 0.001, ^***^p < 0.0001.

Previous studies from colleagues and our team have shown that HIV-infected individuals experience increased systemic microbial translocation even after long-term viral-suppressive ART (Brenchley et al., 2006; Jiang et al., 2009). To study the role of plasma microbiome in HIV disease pathogenesis, we performed microbial 16S sequencing in 40 aviremic ART-treated HIV+ individuals and 51 healthy controls. After removing ASVs with a low abundance and low prevalence from plasma microbiome, we used a filtering factor of 6 to remove potential contaminants and artifacts (Figure 3A); 24 ASVs were retained from 2,997 ASVs (0.80%) in ART-treated HIV+ subjects and healthy controls (Figure 3B). The overall circulating plasma microbial composition was different between HIV-infected individuals and healthy controls (Supplementary Figure S2A; *p* < 0.01). Before filtering, Axis 1 and Axis 2 of the PCoA plot explained 9.2 and 7.2% of the differences within samples, respectively, compared to 37.7 and 15.2%, respectively, after filtering (Supplementary Figure S2A). Next, to analyze the components of the accepted 24 ASVs after filtering, 16 ASVs were significantly different between the two groups using FDR-adjusted values of *p* (Supplementary Table S5). After removing taxa that were not classified at the genus level, HIV-associated enrichment of plasma microbiome included *Veillonella*, *Massilia*, *Haemophilus*, *Arthrobacter*, and *Fusobacterium* genera (Supplementary Figure S2B), the top 3 enriched taxa in HIV+ individuals are shown in Figure 3C. In contrast, HIV-decreased plasma microbiome included *Altererythrobacter, Cryobacterium*, and *Anaerococcus* genera (Supplementary Figures S2B,C).

HIV-associated monocyte activation and chronic inflammation, defined by high levels of pro-inflammatory cytokines, such as IL-1β, IL-6, and TNF-α, play a critical role in disease pathogenesis (Deeks, 2011). To evaluate whether the taxa enriched in plasmas of HIV+ individuals contribute to persistent inflammation in HIV disease, we tested the ability of heat-inactivated bacteria *M. timonae* and *H. parainfluenzae* to induce pro-inflammatory cytokines. Notably, we observed a decrease in *Anaerococcus* sp. in the plasma microbiome of HIV+ individuals compared to controls. *Anaerococcus prevotii* is the commercial for available commensal bacterium within *Anaerococcus* genus which is a commonly isolated flora found on the skin and in the oral cavity (Murphy and Frick, 2013). Therefore, *A. prevotii* was chosen as a negative control bacterium. LPS from *E. coli* 055:B5 was chosen as a positive control. Human PBMCs from 15 healthy individuals were stimulated with *M. timonae*, *H. parainfluenzae*, *A. prevotti*, or LPS for 6 h, and TNF-α, IL-1β, and IL-6 production in monocytes were evaluated using flow cytometry. Treatment of both *M. timonae* and *H. parainfluenzae* but not *A. prevotii* increased the percentages of TNF-α, IL-1β, and IL-6-producing monocytes compared to monocytes in medium controls (*p* < 0.05; Figure 3D). This result suggests that *M. timonae* and *H. parainfluenzae* enriched in the plasmas of HIV+ individuals may play a role in the persistent inflammation in aviremic ART-treated HIV disease.

### Plasma Microbiome in Systemic Lupus Erythematosus

**Figure 4:**
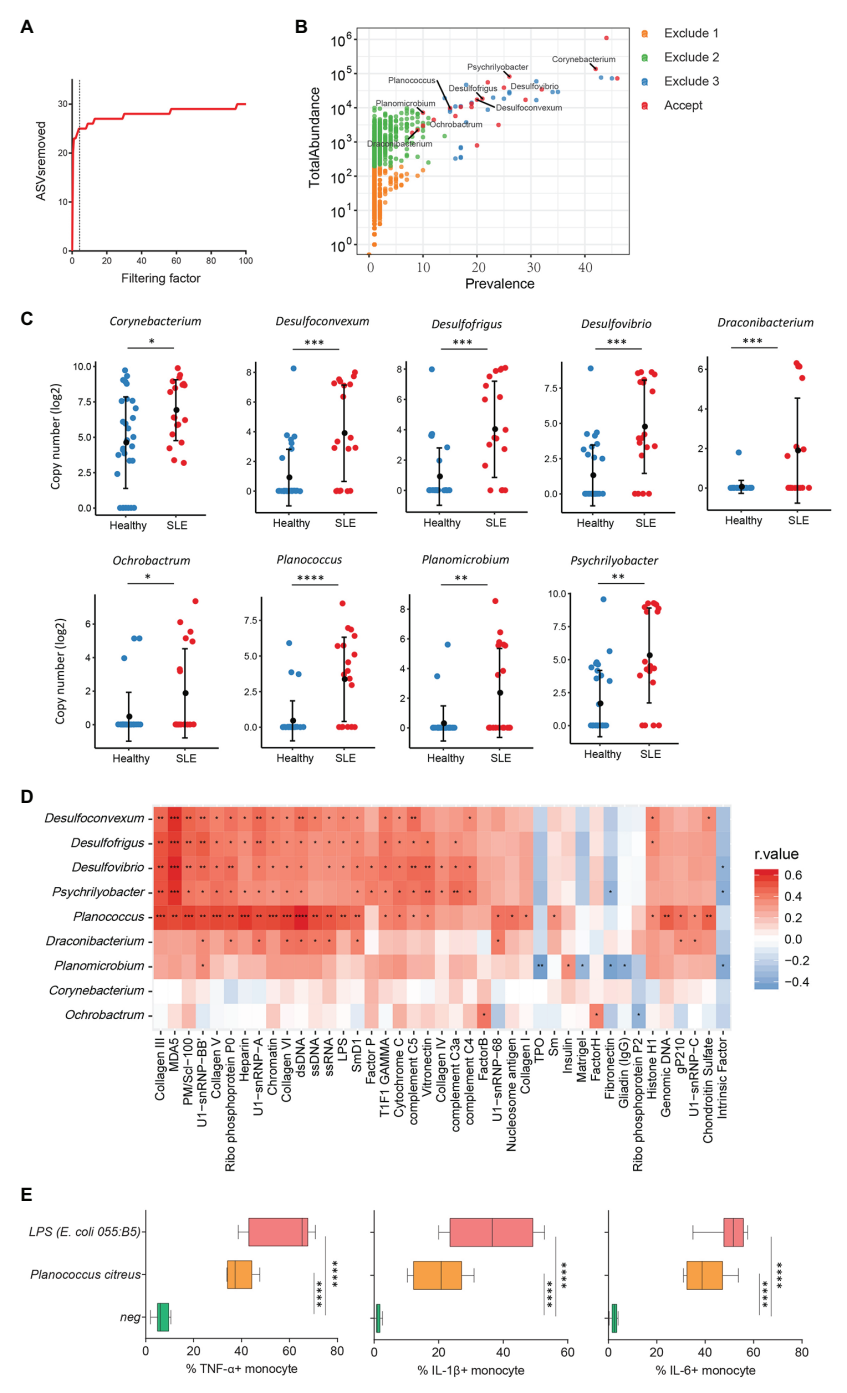
Plasma microbiome in female systemic lupus erythematosus (SLE) patients and healthy female controls. **(A)** The number of removed ASVs with different filtering factors; we chose the filtering factor of 3 which showed the lowest number of the plateau effect. **(B)** The abundance and prevalence of ASVs in each step of plasma microbiome filtration are shown, and the significantly different taxa in SLE patients compared with healthy controls are labeled in black (enriched in SLE patients). **(C)** The significantly different taxa in SLE patients compared with healthy controls. **(D)** Correlations between plasma levels of autoantibodies and differential plasma taxa levels in the two study groups. Correlation coefficient *r* values are indicated by color from red (directly correlations) to blue (inversely correlations); *p*-value significance is shown in the heatmap. **(E)** The percentages of TNF-α, IL-1β, and IL-6-producing monocytes in total monocytes were shown after PBMCs were stimulated with heat-inactivated bacteria (5 × 10^6^ units/ml) or 2 μg/ml of LPS (*n* = 7, healthy individuals). Cytokines were measured in monocytes using flow cytometry after 6 h of stimulation. Box and whisker plots (Min to Max) show the responses of monocytes to each bacterium or LPS. Nonparametric Mann-Whitney’s *U* and Spearman’s rank tests and one-way ANOVA test. ^*^p < 0.05, ^**^p < 0.01, ^***^p < 0.001, ^****^p < 0.0001.

Increasing evidence has revealed a link between increased levels of microbial translocation and autoimmune disease pathogenesis (Fasano, 2012; Mu et al., 2017). To study the association of plasma microbiome and autoimmune disease pathogenesis, 30 healthy women and 19 women with SLE were assessed for the plasma microbiome analysis. All 49 plasma samples were from premenopausal women. After removing low abundance and low prevalence ASVs, a filtering factor of 3 was used to remove potential contaminants (Figure 4A); 21 ASVs were retained from 2,189 ASVs (0.95%) in the healthy controls and SLE patients (Figure 4B). Ten out of the 21 ASVs showed significant differences in SLE patients compared with healthy controls after FDR adjustment, which were enriched in SLE patients (Supplementary Table S6). After removing taxa that were not classified at the genus level, the retained taxa enriched in SLE patients are presented (Figure 4C).

Systemic lupus erythematosus is characterized by loss of tolerance to self-antigens and autoantibody production (Tsokos et al., 2016). To determine the link between plasma microbiome and SLE disease pathogenesis, we examined the association of plasma microbiome with plasma autoantibody reactivities to a panel of 125 autoantigens in SLE patients and healthy controls. Notably, enriched genera of *Desulfoconvexum, Desulfofrigus, Desulfovibrio, Draconibacterium, Planococcus*, and *Psychrilyobacter* in SLE patients were directly correlated with increased plasma levels of various autoantibodies (Figure 4D and Supplementary Table S7), including representative SLE-related IgG autoantibodies, such as anti-double-stranded DNA (anti-dsDNA), anti-nucleosome, anti-histone, and anti-collagen antibodies (Figure 4D; Schett et al., 2002; Reveille, 2004). Most of these microbiome-associated autoantibodies target nuclear and cell-matrix antigens, which are common host self-antigens previously identified in SLE and other autoimmune diseases. These results suggest that alterations of plasma microbiome are associated with autoantibody production in SLE disease. We also tested the ability of heat-inactivated bacteria *Planococcus* to induce pro-inflammatory cytokines. Treatment of *Planococcus citreus* increased the percentages of TNF-α, IL-1β, and IL-6-producing monocytes compared to monocytes in medium controls (*p* < 0.05; Figure 4E). This result suggests that *Planococcus*, related to autoantibody production in SLE, may play a role in the inflammation in SLE disease.

## Discussion

The microbial translocation, including microbiota or microbial fragments, may directly interact with immune cells to remodel immune responses. The blood microbiome has been reported to associate with pathogenesis in a variety of infectious and noninfectious diseases (Clement et al., 2005; Nielsen et al., 2012; Sato et al., 2014; Puri et al., 2018; Poore et al., 2020). However, due to the extremely low levels of microbial biomass in the plasma under physiologic conditions and artifacts during microbial DNA isolation and the sequencing, analysis of blood microbiome is challenging. Although cautions should be taken during the isolation of plasma microbial DNAs to avoid potential contaminations, artifacts from microbial 16S sequencing are still observed and should be controlled in the analysis. In this study, we provided a guideline for filtering out potential contaminations and artifacts in a plasma microbiome analysis and applied to study pathogenesis of various diseases as examples.

Before removing the artifacts from plasma microbiome, we filtered out the ASVs with a low abundance and low prevalence. Extremely low levels of ASVs likely have a minor or nonexistent effect on the immune system. Thus, a 0.005% minimum abundance threshold has been applied in the current study, suggested from a previous study (Bokulich et al., 2013). A larger amount of ASVs with a low abundance across the samples may be a result of microbial or microbial product translocation across a permeable mucosa (e.g., gut). ASVs with a low abundance and low prevalence were removed, and only ASVs with a prevalence of more than 1/3 in any study group were retained. However, the distinct species and taxa may play similar functions in the individual microbiome. Therefore, taxa unique to individuals may play similar roles in disease pathogenesis. Removing any sequence that is not in at least 1/3 of the cohort may also filter out some meaningful taxa related to the disease. To exclude the potential artifacts, controls are critical because they reflect contaminations during the process of blood collection until 16S sequencing, the taxa detected in the water controls should be removed from the experimental samples at the ASV levels. However, the removal of ASVs should be performed with cautions, because some ASVs present in the controls may originate from cross-contamination with the experimental samples during DNA isolation, PCR, or sequencing. Due to the lack of plasma 16S templates available to compete for amplification reagents in the PCR and sequencing stages, the background or contaminant ASVs in each control likely will have a similar or higher copy number than those in the plasma samples. In this case, a filtering factor was applied to remove potential contaminants from plasma microbiome, whereby we only remove ASVs with abundance in experimental samples with less than a factor of times of the corresponding abundance in the water controls. The choice of this filtering factor is a processing choice for which we suggest as a rule of thumb to examine the filtering plateau effect and to make a determination regarding which ASVs may be retained. Using this filtering strategy, we observed that 97–99% of the ASVs in the plasma microbiome data were accounting for extremely low abundance, low prevalence, contamination, or artifacts and should be excluded in analyses.

Next, we performed plasma microbiome analyses in tobacco-smoking individuals as an example. Smoking has been associated with an altered oral microbiota and periodontal diseases (Mason et al., 2015; Yu et al., 2017), which may facilitate the translocation of some oral microbiota into the blood. Consistent with a previous study (Wu et al., 2016), we discovered an enrichment of *Streptococcus* in the saliva microbiota from smokers compared to non-smokers (Figure 2G). Importantly, *Streptococcus* was also found enriched in the plasma microbiome from smokers compared to non-smokers (Figure 2F). Although many other taxa were enriched in the saliva of smokers, they were not enriched in the paired plasma samples, suggesting that *Streptococcus* may have a stronger migration ability from smoking-associated oral mucosa to the circulation. *Streptococcus* in the blood has been found to play a role in atherosclerosis and cardiovascular diseases (Dos Reis et al., 1982; Brown et al., 2014; Jackson et al., 2016).

We also analyzed plasma microbiome in HIV and SLE as well as the link between plasma microbiome and disease pathogenesis. After the implementation of our filtration guidelines to remove the potential contaminants and ASVs with a low abundance, we noted significant differences in the *β*-diversity and composition of plasma microbiome between HIV+ subjects and healthy controls. Most of the taxa enriched in HIV+ individuals in this study are well-known pathogenic bacteria. *Arthrobacter* spp. and *M. timonae* have been isolated from blood, cerebrospinal fluid, and bone of clinical patients with infectious diseases or end-organ diseases (Lindquist et al., 2003; Mages et al., 2008). Moreover, *H. parainfluenzae* is an opportunistic pathogen (Smith et al., 1976); we found that *in vitro* treatment with HIV-enriched heat-inactivated *M. timonae* and *H. parainfluenzae* induced robust inflammatory responses in human monocytes (Figure 3D). SLE is a systemic autoimmune disease, and autoantibodies play a key role in disease pathogenesis. However, the underlying etiology and mechanisms of autoantibody production in SLE are not fully understood (Graham and Utz, 2005). Persistent immune activation induced by the translocation of microbial components from the gastrointestinal tract or other mucosal sites into the circulation has been considered one of the predisposing factors in SLE (Brenchley et al., 2006; Brenchley and Douek, 2012; Ogunrinde et al., 2019). In this study, we found that some SLE-enriched taxa had a direct correlation with plasma autoantibody levels (Figure 4D). The functional evaluation of these SLE-enriched taxa in lupus disease merits further investigation.

## Conclusion

In summary, we provide a strategy for filtering potential contaminants and backgrounds to analyze plasma microbiome, which provides a promising method to study translocated bacteria or bacteria in tissues in various diseases and their roles in modulating systemic immune responses and disease pathogenesis.

## Data Availability Statement

The full plasma microbiome sequencing data sets can be available at https://www.ncbi.nlm.nih.gov/sra/PRJNA551477.

## Ethics Statement

The studies involving human participants were reviewed and approved by Volunteers with SLE, HIV-infected individuals and healthy individuals were recruited from the Medical University of South Carolina (MUSC) Lupus Clinic, Clinic of Infectious Diseases, and MUSC campus. This study was approved by each participating institutional review boards. All participants provided written informed consent. The patients/participants provided their written informed consent to participate in this study.

## Author Contributions

ZLu and WJ designed the project. ZLu analyzed plasma microbiome next-generation sequencing data and wrote the manuscript. ZLu, AA, and ML contributed to data interpretation and discussion. ZLu, EO, Q-ZL, ML, LH, DK, and JO performed experiments. WJ, AA, EO, Q-ZL, BT, ZLi, and GG contributed to the study design and revised the manuscript. All authors contributed to the article and approved the submitted version.

### Conflict of Interest

The authors declare that the research was conducted in the absence of any commercial or financial relationships that could be construed as a potential conflict of interest.

## Footnotes

^1^https://qiime2.org/
