# Supplementary figures and images for: Rigorous Plasma Microbiome Analysis Method Enables Disease Association Discovery in Clinic
Source: Front Microbiol. 2021 Jan 8;11:613268. doi: 10.3389/fmicb.2020.613268 (PMC7820181; doi:10.3389/fmicb.2020.613268)

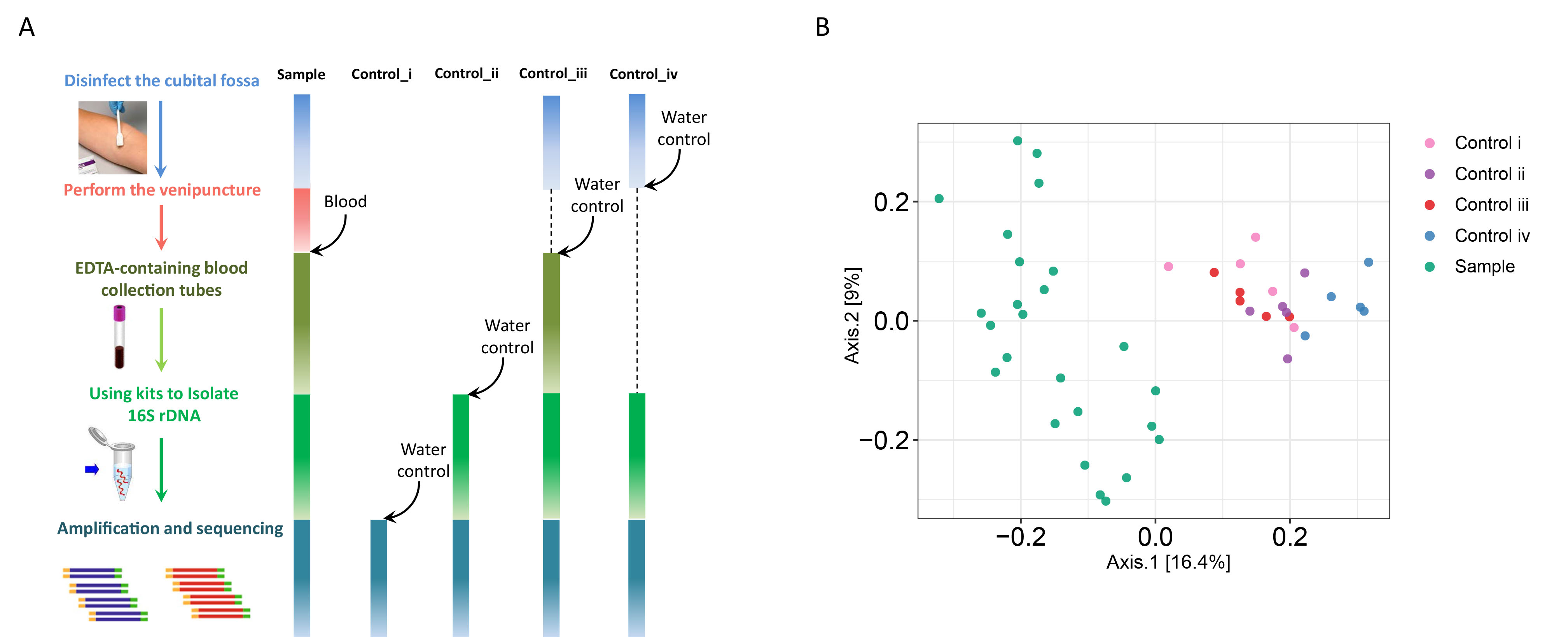

Supplement: Supplementary file 1 [file Figure_S1.jpeg]

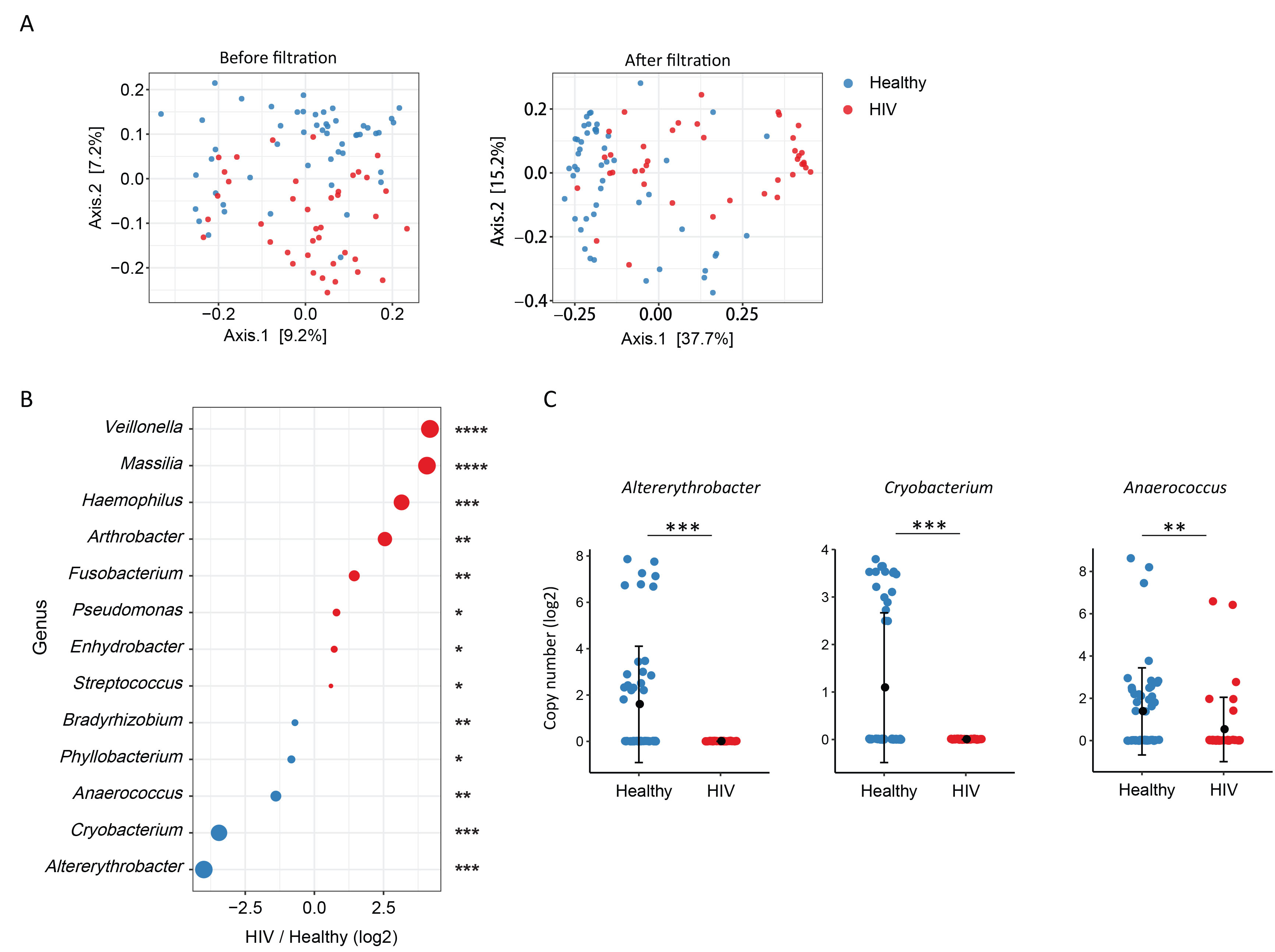

Supplement: Supplementary file 2 [file Figure_S2.jpeg]
